# Analysis of Abnormal Intra-QRS Potentials in Signal-Averaged Electrocardiograms Using a Radial Basis Function Neural Network

**DOI:** 10.3390/s16101580

**Published:** 2016-09-27

**Authors:** Chun-Cheng Lin

**Affiliations:** Department of Electrical Engineering, National Chin-Yi University of Technology, Taichung 41170, Taiwan; cclin@ncut.edu.tw; Tel.: +886-4-2392-4505 (ext. 7238)

**Keywords:** abnormal intra-QRS potentials, ventricular late potentials, radial basis function neural network, orthogonal least squares, ventricular tachycardia

## Abstract

Abnormal intra-QRS potentials (AIQPs) are commonly observed in patients at high risk for ventricular tachycardia. We present a method for approximating a measured QRS complex using a non-linear neural network with all radial basis functions having the same smoothness. We extracted the high frequency, but low amplitude intra-QRS potentials using the approximation error to identify possible ventricular tachycardia. With a specified number of neurons, we performed an orthogonal least squares algorithm to determine the center of each Gaussian radial basis function. We found that the AIQP estimation error arising from part of the normal QRS complex could cause clinicians to misjudge patients with ventricular tachycardia. Our results also show that it is possible to correct this misjudgment by combining multiple AIQP parameters estimated using various spread parameters and numbers of neurons. Clinical trials demonstrate that higher AIQP-to-QRS ratios in the X, Y and Z leads are visible in patients with ventricular tachycardia than in normal subjects. A linear combination of 60 AIQP-to-QRS ratios can achieve 100% specificity, 90% sensitivity, and 95.8% total prediction accuracy for diagnosing ventricular tachycardia.

## 1. Introduction

As a noninvasive diagnostic tool for detecting ventricular arrhythmia, a signal-averaged electrocardiogram can be employed to detect low amplitude, high frequency ventricular late potentials (VLPs) at the end of a QRS complex, or abnormal intra-QRS potentials (AIQPs). VLPs have been reliably applied to assessing risk among VT patients recovering from myocardial infarction, diagnosing patients who are unconsciousness for unknown reasons, and detecting ischemic heart diseases [1]. They have also been applied to diagnosing patients with thalassemia [2], epilepsy [3], cardiac sarcoidosis [4] and arrhythmogenic right ventricular cardiomyopathy [5]. Although time-domain VLP analysis has confirmed its high negative predictive value, it lacks a sufficient positive value to diagnose a person as being ill [2]. There have been a number of studies published on methods for improving VLP detection, e.g., spectral turbulence [6], the wavelet transform-based method [7] and principal component analysis [8], however, no consensus exists yet on their methods and clinical applications.

VLP is a non-invasive approach to detecting the presence of an arrhythmic substrate. Featuring low conduction, the presence of an arrhythmic substrate leads to a reentrant excitation, and may even cause lethal ventricular arrhythmia, including ventricular tachycardia (VT) and ventricular fibrillation [1]. From a cardiac electrophysiological point of view, the presence of VLP reflects delayed excitation and fragmented electrical activity as a consequence of low conduction in the cardiac muscles. Several studies have pointed out that a QRS complex has extended duration owing to the delay in the conduction of electrical activity. Although a reentrant excitation event, caused by an arrhythmic substrate, is not accompanied by VLP in all instances, it is likely to appear as part of AIQP in a QRS complex [9,10]. As an alternative form of VLP, AIQP is able to completely reflect a reentrant excitation due to an arrhythmic substrate, and may improve the diagnostic performance of a signal-averaged electrocardiogram.

Extracting AIQPs is a challenging task, since they are extremely weak notch and slur signals with abrupt changes in slope, embedded in a QRS complex. Moreover, AIQPs are essentially random signals that differ among VT patients. As presented by Gomis et al. [11] and Lander et al. [12], AIQPs are estimated in a discrete-time cosine transform (DCT)-domain autoregressive moving average (ARMA) model as the residual signal, that is, the difference between a target QRS complex and a synthesized one. One remarkable advantage is that the AIQP of interest can be separated from a normal QRS complex by means of a low order ARMA model. This approach can achieve a total prediction accuracy (TPA) of between 63.6% and 75.8% for diagnosing VT patients, provided that either X, Y or Z lead AIQP parameters are employed. In contrast, a linear prediction model may be employed to extract the signals with abrupt slope change embedded in a QRS complex [13,14]; these are referred to as the unpredictable intra-QRS potentials (UIQPs). The analysis of UIQPs has been validated experimentally as an effective approach to identifying the presence of AIQPs, and accordingly, as a way to diagnose VT patients. Use of either X, Y or Z lead UIQP parameters provided a TPA between 76.4% and 83.3% in one study [13], and a TPA between 72.2% and 79.2% in another [14]. A wavelet transform-based approach has been proposed by Tsutsumi et al. [15] and Yodogawa et al. [16] as a way to analyze the high frequency components of a QRS complex, and to diagnose patients with lethal ventricular arrhythmias. Specificity as high as 79.4%–93.8%, but sensitivity as low as 23.2%–37.2% were found in patients with lethal ventricular arrhythmias [15], whereas another study showed sensitivity up to 96%, but specificity of 64.3%, were seen among patients with VT or ventricular fibrillation [16].

As presented in a great number of previous studies, a skillful combination of AIQP and VLP parameters can further improve diagnostic accuracy for patients at high risk of ventricular arrhythmia, but improved accuracy of the AIQP estimates is required for clinical application. Previously, most AIQP estimates were made with linear models or by means of linear transformation. It may be better to model a QRS complex as a nonlinear signal, due to the elaborate working mechanism of a human heart. To estimate AIQPs, our previous study [17] employed a nonlinear radial basis function neural network (RBFNN), as commonly seen in the disciplines of function approximation [18] and data classification [19]. In this manner, a strong, slow varying, normal QRS complex is synthesized by an RBF neural network, and an approximation error is regarded as the weak, rapidly varying AIQPs. Short of clinical trials, a great amount of neurons are employed to build an RBF neural network, and AIQPs are modeled as white noise [17]. This work is an improved version of our prior research, applied to diagnosing patients with VT.

Nevertheless, it is a non-trivial and challenging task to precisely extract the AIQP of interest from a QRS complex, because the approximation error may not merely include the wanted contribution from the AIQP, it may also comprise the unintended contribution from part of the normal QRS complex. One major problem is that a decrease in the approximation error may occur in the event that both contributions are out of phase, which could lead a clinician to misjudge the presence of AIQP. There is no way to further extract the AIQP estimation error caused by part of the normal QRS complex, since in practical situations, AIQPs are unpredictable and random signals. In this study, we conducted an in-depth investigation into the AIQP estimation error with AIQPs simulated as multiple noise sources, and found improved accuracy in clinical trials using linear combinations of the AIQP parameters. In short, the aim of this work is: (1) to develop a systematic RBF neural network-based approach for AIQP estimation from a measured QRS complex, as a high performance diagnostic tool for VT patients; (2) to evaluate the effects of AIQP estimation errors caused by part of the normal QRS complex; and (3) to improve the diagnostic accuracy by taking a linear combination of AIQP parameters.

The remainder of this paper is outlined as follows: Section 2 describes the study subjects and the basics of this RBF neural network-based analysis of AIQP, Section 3 details the results from simulations and clinical trial, Section 4 presents a discussion of the findings, and Section 5 concludes the paper.

## 2. Materials and Methods

### 2.1. Data Acquisition

The Ethics Committee of Taipei Jen-Chi General Hospital approved the study and each patient provided informed consent. As in previous studies [13,14], we categorized subjects into a normal group and a VT patient group for comparison purposes. The normal group consisted of 20 men and 22 women, all Taiwanese adults aged 58 ± 14. All healthy subjects had a normal clinical history, physical examination, 12-lead ECG, and echocardiogram. The VT group was composed of 30 VT patients, all with sustained VT documented by 24-h Holter ECG monitoring. The VT patients all suffered from chronic ischemic heart disease and had survived clinically documented myocardial infarction.

All subjects were supine for the high resolution electrocardiograms. Using a Megacart^®^ recorder (Siemens-Elema, Solna, Sweden) equipped with a bipolar XYZ lead system, we recorded ECG waveforms over a duration of 10 minutes at a sampling frequency of 2 kHz and with 12-bit quantization, and then saved them on a hard drive for subsequent signal processing and analysis. As recommended in the 1996 American College of Cardiology Expert (ACCE) Consensus Document [1], signal-averaged electrocardiograms are employed as a standard approach for reducing the random noise effect on the signal of interest. Taking advantage of a 40–250 Hz bidirectional Butterworth filter, we kept the noise level contained in the processed signal-averaged electrocardiogram below 0.7 µV. Following recommendations by the ACCE [1], we employed vector magnitude analysis to determine both the onset and the offset of a QRS complex. We used three standardized time-domain VLP parameters to detect VLP, namely the filtered QRS duration (fQRSD), the root-mean-square (RMS) voltage of a QRS complex in the last 40 ms (RMS40), and the duration of a low amplitude signal below 40 mV (LAS40).

### 2.2. RBF Neural Network for the AIQP Analysis

As described by Haykin [18], an RBF neural network refers to a network built with multiple RBFs using a curve fitting approach. An RBF neural network involves an input layer, a hidden layer and an output layer. The input layer is basically an interface between the inputs and a network. The hidden layer, consisting of multiple neurons, each representing an RBF, takes charge of nonlinear mapping. A neuron’s output decreases steeply with the distance between its input and the RBF center. Subsequently, an output in the output layer is generated by taking a linear combination of the outputs in the hidden layer.

**Figure 1:**
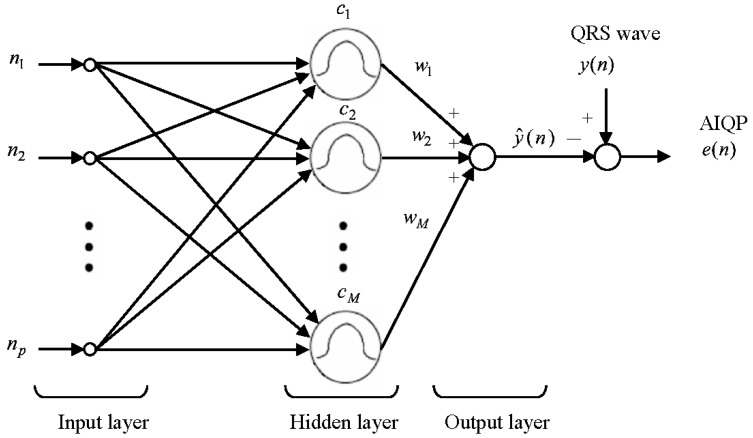
Block diagram of an RBF neural network for the AIQP analysis.

As illustrated in Figure 1, in an RBF neural network built for analysis of AIQP, there are *p* inputs in the input layer, *M* neurons in the hidden layer and a single output in the output layer. The input of the input layer is represented as a *p*-dimensional discrete time variable *n* = {*n*_1_,*n*_2_,…,*n_p_*} = {1,2,…,*p*}, where *p* denotes the length of the QRS complex. The aim of the RBF neural network is to synthesize the QRS complex, while there is an error, referred to as an approximation error, between the synthesized QRS complex and the target one. The approximation error is treated as the AIQP of interest. A Gaussian RBF is defined as:

(1) $$\phi (n-c)=\exp\left(-\frac{{\left(n-c\right)}^{2}}{2\sigma^{2}}\right),\;c\geq 0,$$

where *c* and *σ* represent the center and the spread parameter of the RBF, respectively. Taking the RBF transform on the input, the output *Z_j_* of the *j*th neuron in the hidden layer is given as:

(2) $$z_{j}(n)=\phi (n-c_{j}),\;j=1,2,\cdots ,M,$$

where *c_j_* denotes the center of the *j*th neuron, and *M* the number of neurons in the hidden layer. By taking a linear combination of *z_j_*, *j* = 1,2,…,*M*, an output in the output layer is generated as:

(3) $$\hat{y}(n)=\sum_{j=1}^{M}w_{j}z_{j}(n),$$

where *w_j_* signifies the weight of *z_j_*. A back substitution of Equation (2) into Equation (3) yields:

(4) $$\hat{y}(n)=\sum_{j=1}^{M}w_{j}\phi (n-c_{j}),$$

Hence, a target output of an RBF neural network can be expressed as:

(5) $$y(n)=\hat{y}(n)+e(n)=\sum_{j=1}^{M}w_{j}\phi (n-c_{j})+e(n),$$

where *e*(*n*) represents the approximation error. Alternatively, it can be represented in matrix form as:

(6) $$\left[\begin{array}{c} y(1)\\ y(2)\\ \vdots \\ y(p) \end{array}\right]=\left[\begin{array}{ccc} \phi (1-c_{1}) & \cdots & \phi (1-c_{M})\\ \phi (2-c_{1}) & \cdots & \phi (2-c_{M})\\ \vdots & \vdots & \vdots \\ \phi (p-c_{1}) & \cdots & \phi (p-c_{M}) \end{array}\right]\left[\begin{array}{c} w_{1}\\ w_{2}\\ \vdots \\ w_{M} \end{array}\right]+\left[\begin{array}{c} e(1)\\ e(2)\\ \vdots \\ e(p) \end{array}\right],$$

that is:

(7) y=ΦW+e,

where **Φ** and **W** are referred to as an RBF matrix and a weight vector, respectively. Given the number of neurons *M*, the spread parameter and the center location of each RBF, the optimal weight vector **W**_o_ can be found by minimizing the sum of squared errors, defined as:

(8) $$\xi =\sum_{n=1}^{p}e^{2}(n)=\sum_{n=1}^{p}{\left(y(n)-\hat{y}(n)\right)}^{2},$$

or in a compact form as:

(9) $$\xi =e^{T}e,$$

where *T* represents a transpose operator. Substituting Equation (7) into Equation (9) and letting $\frac{\partial \xi }{\partial \omega_{j}}=0,$ (*j* = 1,2,…,*M*) gives:

(10) $$\Phi^{T}y-\Phi^{T}\Phi W=0,$$

Accordingly, the optimal weight vector is found as:

(11) $$W_{o}={\left(\Phi^{T}\Phi \right)}^{-1}\Phi^{T}y=\Phi^{+}y,$$

and the neural network output is evaluated as:

(12) $${\hat{y}}_{o}=\Phi W_{o}=\Phi \Phi^{+}y,$$

### 2.3. RBF Center Locations Determined through an Orthogonal Least Squares Algorithm

As a prerequisite for building an RBF neural network, it’s necessary to specify the number of neurons *M* in the hidden layer, the spread parameter and the center location of each RBF. It is known that an adequate number of neurons gives rise to a more accurate approximate QRS complex, but this might overestimate the normal components contained in a QRS complex and underestimate the AIQP instead. The shape parameters of an RBF, including the width and the smoothness, are reflected by the spread parameter, according to which a widened RBF is employed to depict the low frequency components contained in a QRS complex, and a sharp one is to describe the high frequency components. In this study, the same spread parameter is shared by all the RBFs employed, meaning that they are identically shaped. In this context, the approximation error can be treated as the frequency components higher than RBFs, and may then offer a way to estimate the embedded AIQP. We employed various numbers of neurons and distinct spread parameters to construct a number of RBF neural networks to investigate their influence on the accuracy of AIQP estimates and the diagnostic accuracy for VT patients.

Subsequently, each RBF center is in turn determined through an orthogonal least squares algorithm as presented in Chen et al. [20], where all the locations of inputs in the input layer are treated as candidate centers, of which the one with the maximal error reduction ratio is selected as an added center. For instance, the *L*th RBF center is selected from the locations of the remaining *p*-*L* + 1 inputs, where *p* represents the number of inputs and *L* − 1 the number of RBF centers already determined using the orthogonal least squares algorithm. A step-by-step approach is provided as follows. Using Gram-Schmidt orthogonalization, an RBF matrix **Φ** is decomposed into:

(13) $$\Phi =\left[\begin{array}{cccc} \phi_{1} & \phi_{2} & \cdots & \phi_{L} \end{array}\right]=\left[\begin{array}{cccc} s_{1} & s_{2} & \cdots & s_{L} \end{array}\right]\;\left[\begin{array}{cccc} 1 & a_{12} & \cdots & a_{1L}\\ 0 & 1 & \cdots & a_{2L}\\ \vdots & \vdots & \vdots & \vdots \\ 0 & 0 & \cdots & 1 \end{array}\right]=SA,$$

where $\phi_{L}$ denotes an RBF vector corresponding to a candidate RBF center, **A** an *L* × *L* upper triangular matrix, **S** a matrix of dimension *p* × *L*, and **s***_i_*, *i* = 1, …, *L*, orthogonal vectors such that:

(14) $$S^{T}S=H,$$

where **H** represents a positive diagonal matrix with diagonal elements given as:

(15) $$h_{i}=s_{i}^{T}s_{i},$$

A Gram-Schmidt orthogonization process is described as follows:

(16) $$s_{1}=\varphi_{1},$$

(17) $$\alpha_{ik}=\frac{s_{i}^{T}\phi_{k}}{s_{i}^{T}s_{i}},\;1\leq i\leq k,$$

(18) $$s_{k}=\phi_{k}-\sum_{i=1}^{k-1}\alpha_{ik}s_{i}.$$

Substitution of Equation (13) into Equation (7) gives:

(19) y=ΦW+e=SAW+e=SG+e,

where **G** = **AW**. In this fashion, a target signal is expressed as a linear combination of orthogonal basis vectors **s***_i_*. According to the least squares approach, the optimal weight vector **G*_o_*** is represented as:

(20) $$G_{o}=\left[\begin{array}{c} g_{o,1}\\ g_{o,2}\\ \vdots \\ g_{o,M} \end{array}\right]={(S^{T}S)}^{-1}S^{T}y=S^{*}y=H^{-1}S^{T}y,$$

where:

(21) $$g_{o,i}=\frac{s_{i}^{T}y}{s_{i}^{T}s_{i}},$$

Since **S** is a matrix composed of orthogonal basis functions, the square of the target signal is expressed as:

(22) $$y^{T}y=G^{T}S^{T}SG+e^{T}e=\sum_{i=1}^{L}g_{i}^{2}h_{i}+e^{T}e.$$

The error reduction ratio of the *L*th RBF center is defined as:

(23) $${\left[err\right]}_{L}=\frac{g_{0,L}^{2}h_{L}}{y^{T}y}.$$

Equation (23) evaluates the error reduction ratios of all the remaining candidate centers; of these, the one with the maximal error reduction ratio is seen as the *L*th RBF center. The above step-by-step approach is repeated until a total of *M* RBF centers are determined. A substitution of all the RBFs into Equation (11) yields the optimal weight vector **W***_o_*, and Equation (12) gives the output signal ${\hat{y}}_{o}$ and the approximation error $e_{o}=y-{\hat{y}}_{o}$.

### 2.4. Definition of AIQP

In this study, we expected that most of a QRS complex could be synthesized by an RBF neural network; for this reason, the approximation error between the output and a target signal is treated as the embedded AIQP for VT diagnosis. As a quantitative measure, AIQP is defined as the root mean square value of the approximation error over a cycle of QRS complex, that is:

(24) $$\mathrm{AIQP}\text{\_}l(M,\sigma )=\sqrt{\frac{1}{p}\sum_{n=1}^{p}e_{o}^{2}(n)},$$

where *l* represents the X, Y or Z lead, *M* the number of neurons, *σ* the spread parameter of the RBF, *p* the period of the QRS complex, and *e_o_*(*n*) the approximation error gained through the least square approach. Furthermore, the AIQP-to-QRS (AQR) ratio is defined as the ratio between the estimated AIQP and the root-mean-square value of the QRS complex, formulated as:

(25) $$\mathrm{AQR}\text{\_}l(M,\sigma )=\mathrm{AIQP}\_l/\sqrt{\frac{1}{p}\sum_{n=1}^{p}y^{2}(n)},$$

where *y*(*n*) denotes a target signal, namely a measured QRS complex. In comparison, AIQP is more likely to be observed among VT patients than among normal subjects.

### 2.5. Statistical Approaches

All statistical analyses were undertaken using the Statistical Package for the Social Sciences^®^. (SPSS 11.0, SPSS Inc., Chicago, IL, USA). An *F*-test was performed to compare the variances of different variables, and a two-tailed Student’s *t*-test was adopted to compare the means of two independent variables. Statistical significance was defined as *p* < 0.05. We used a Fisher’s linear discriminant analysis to combine the time-domain VLP and AIQP parameters and to classify the subjects into the normal and the VT groups. The details of the method have been described in our previous study [14]. Three local performance indices, including the specificity, the sensitivity and the total prediction accuracy (TPA) [21], were calculated to evaluate the accuracy of diagnosing the VT patients. A receiver operating characteristic curve was applied to analyze the global diagnostic performance, and used the area under the receiver operating characteristic curve (AUC) as a measure of global performance [22,23].

## 3. Results

### 3.1. Simulation Results

**Figure 2:**
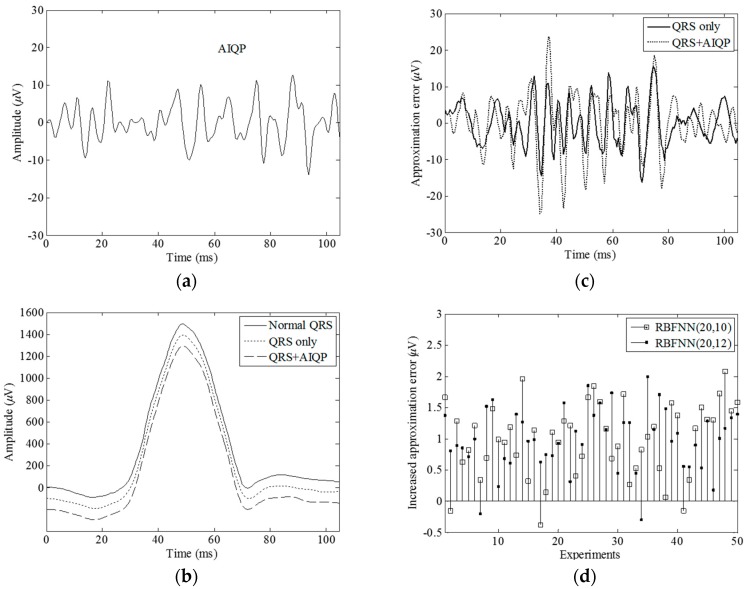
Simulation results for a normal QRS complex in the presence and absence of a simulated AIQP: (**a**) an AIQP simulated as 40–250 Hz colored noise; (**b**) a normal QRS complex (solid line) simulated as an X-lead QRS complex of a normal subject, the approximation outputs in the absence (dotted line) and the presence (dashed line) of AIQP using an RBFNN (20, 10); (**c**) the approximation errors in the presence (dotted line) and the absence of (solid line) AIQP using the RBFNN(20, 10); and (**d**) the increase in the approximation errors using the RBFNN(20, 10) and an RBFNN(20, 12). RBFNN (*M*, *σ*) denotes a radial basis function neural network with *M* neurons and a spread parameter of *σ.*

We expected that a normal QRS complex could be synthesized by an RBF neural network, and low amplitude, high frequency AIQP would account for the observed approximation error. In simple terms, there exists a higher level of approximation error in the presence of AIQP. We used simulation and experiments to demonstrate the superiority of this RBF neural network for detecting AIQP. Because of the randomness of the AIQP, it was simulated as a 40–250 Hz colored noise and was generated as filtered white noise through a fourth order band pass Butterworth filter between 40 and 250 Hz. Presented in Figure 2a is a simulated AIQP with an root-mean-square value of 5 µV. Figure 2b shows: (i) a simulated normal QRS complex with an root-mean-square value of 620.6 µV plotted as a solid line, from the X-lead signal-averaged electrocardiogram of a normal subject; (ii) an output waveform plotted as a dotted line and designated as the “QRS only” case in the absence of AIQP, synthesized by an RBF neural network with 20 neurons and a spread parameter of 10, abbreviated as RBFNN(20, 10); and (iii) an output in the presence of AIQP plotted as a dashed line and designated as the “QRS + AIQP” case. It is worth noting that the output of an RBF neural network can closely approximate the normal QRS complex. Demonstrated in Figure 2c are the approximation errors in the “QRS only” case, plotted as a solid line, and in the “QRS + AIQP” case, plotted as a dotted line. We observed that there was an approximation error in any case, that is, a 6.0 µV RMS error in the “QRS only” case, and 8.0 µV RMS error in the “QRS + AIQP” case. As expected, the addition of AIQP to a QRS complex led to a rise in the approximation error.

As revealed in Figure 2c, it is worth mentioning that an approximation error attributed to the addition of AIQP and to an inherent error found in the “QRS only” case. Very likely, the addition of AIQP increases (decreases) the level of approximation error provided that it is mostly in (out of) phase with the inherent error because of the randomness of AIQP. A drop in the approximation error in this case will lead to a misjudgment. In order to demonstrate the issue caused by the inherent error, we conducted 50 repetitions of AIQP estimates in the “QRS + AIQP” case, each with a colored random noise having an root-mean-square value of 5 V and a bandwidth between 40 and 250 Hz added to the normal QRS complex. Figure 2d shows the comparison between the respective increases in the approximation error by an RFBNN(20, 10) and an RFBNN(20, 12). It is found that the increases range between −1.05 and 2.69 µV through all of the repetitions, although the same amplitude and bandwidth colored noise is added to the normal QRS complex. Yet, as can also be seen in Figure 2d, there are a number of inconsistencies in the AIQP estimates between two such neural networks. For instance, in repetition 46, a small increase of 0.18 µV was seen in the RBFNN(20, 12) case, while an increase of up to 1.73 µV was observed in its counterpart. More importantly, there were three occasions on which the presence of AIQP actually diminished the output level in the RBFNN(20, 10) case, and two occasions in the RBFNN(20, 12) case, meaning that there were a total of five misjudgments among all the observations. Nevertheless, reflecting on this contradiction led to the notion that the three misjudgments by RBFNN(20, 10) could be corrected by RBFNN(20, 12), and vice versa, and the two by RBFNN(20, 12) could be corrected by RBFNN(20, 10).

**Figure 3:**
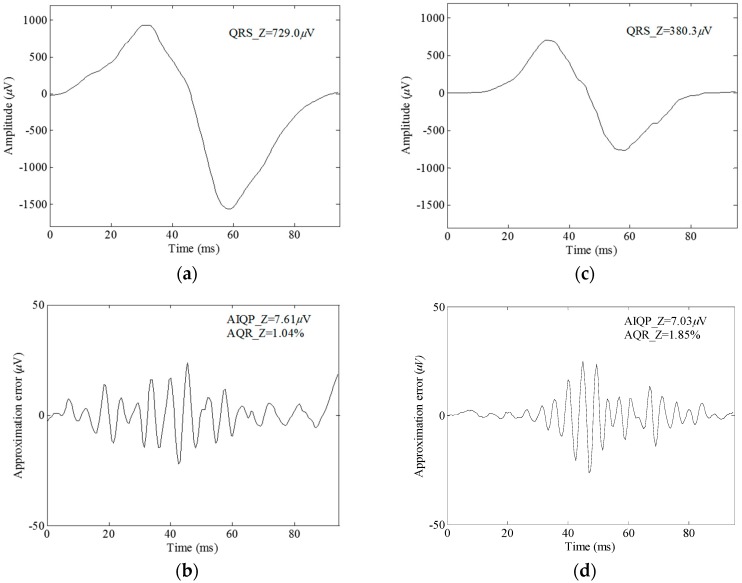
Comparison of the Z-lead QRS waves and approximation errors using an RBFNN(20, 10), (**a**) the QRS wave and (**b**) the approximation error in a normal subject case; and (**c**) the QRS wave and (**d**) the approximation error in a VT patient case.

An RBF neural network is known as a nonlinear system, but the approximation error is in proportion to the strength of the QRS complex. For instance, the root-mean-square value of the normal QRS complex in Figure 2b doubled to 12 µV in the absence of AIQP when the complex was multiplied by a factor of 2. Hence it is likely that scaling a QRS complex affects the AIQP estimates when making a comparison between a normal subject and a VT patient. For this reason, we here define a quantitative parameter AQR. Shown in Figure 3a,c are a 729.0 µV RMS Z-lead QRS complex of a normal subject and a 380.3 µV RMS of a VT patient, respectively. Unexpected approximation errors of 7.61 and 7.03 µV are seen in Figure 3b, the normal subject case, and in Figure 3d, the VT patient case, respectively. Thus, there is no way to accurately tell a VT patient from a normal subject by means of a single parameter, i.e., the approximation error. Alternatively, these misjudgments can be corrected by comparison of another parameter, AQR, since there is an AQR of 1.85% in the VT patient case, which is higher than 1.04% in the normal subject.

### 3.2. Clinical Trial Results

**Figure 4:**
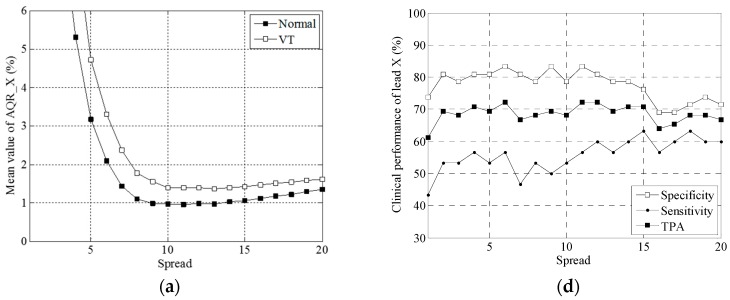

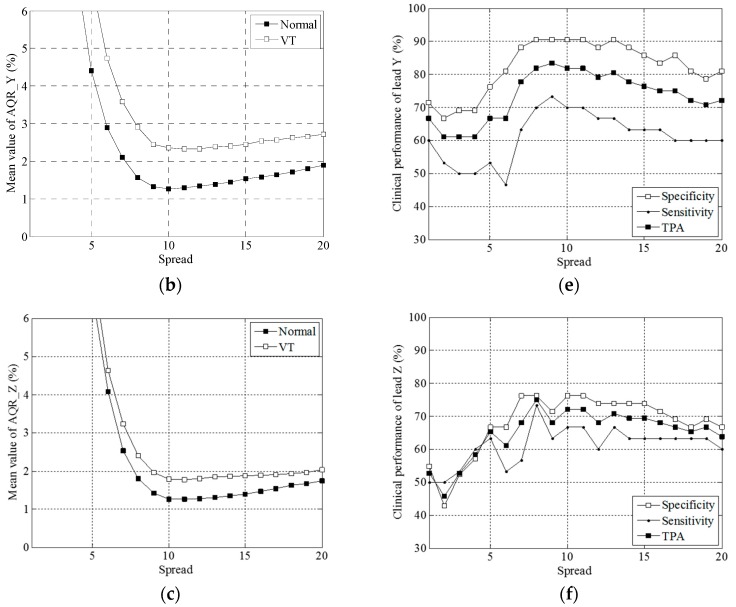
Graphs of the mean AQR vs. the spread parameter in leads (**a**) X; (**b**) Y and (**c**) Z and of the clinical performance vs. the spread parameter in leads (**d**) X; (**e**) Y and (**f**) Z, keeping the neuron number fixed at 10.

The number of neurons *M* and the spread parameter *σ* in an RBF neural network are found to dominate the accuracy of VT diagnosis. Presented in Figure 4 are comparisons of the mean AQR values and plots of clinical performance, i.e., specificity, sensitivity and total prediction accuracy, in leads X, Y and Z, versus the spread parameter. As indicated in Figure 4a–c, the VT case had a higher mean AQR value than the normal case in leads X, Y and Z with a fixed number of neurons (namely 20) in the RBF neural network. The minimal value occurs at *σ* = 10.

**Figure 5:**
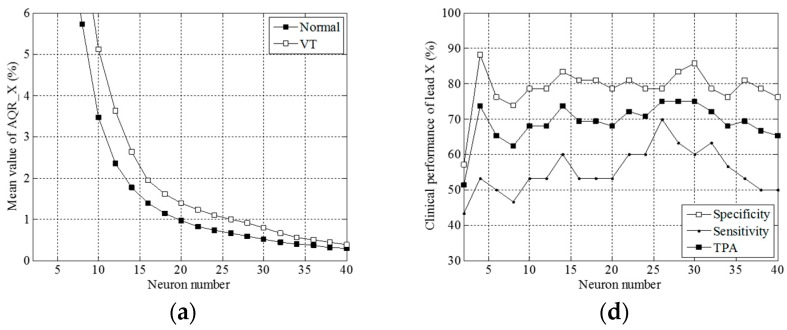

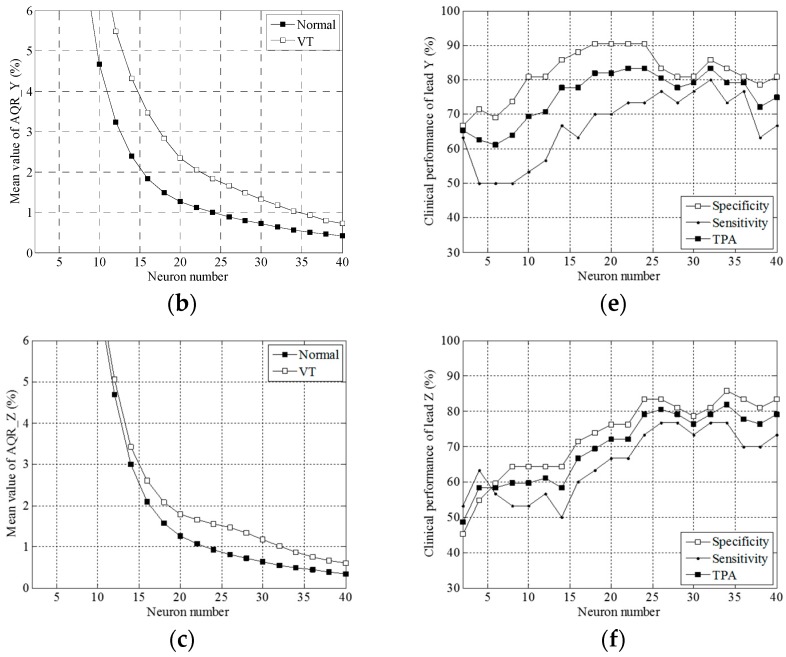
Graphs of the mean AQR vs. the neuron number in leads (**a**) X; (**b**) Y and (**c**) Z, and also, graphs of the clinical performance vs. the spread parameter in leads (**d**) X; (**e**) Y and (**f**) Z, keeping the spread parameter fixed at 20.

Exhibited in Figure 4d–f are plots of the local performance indices against the spread parameter in the X, Y and Z leads. TPA is well maintained at approximately 70% between *σ* = 2 and *σ* = 15 in the X lead, at approximately 80% between 7 and 14 in the Y, and at approximately 70% between 7 and 16 in the Z lead. Both TPA and sensitivity reached their maximum at *σ* = 12, nine and eight in the X, Y and Z leads, respectively. As can be found in Figure 5a–c, all the mean values of AQR decrease with the number of neurons *M*, and a higher level appears in the VT than in the normal case.

**Figure 6:**
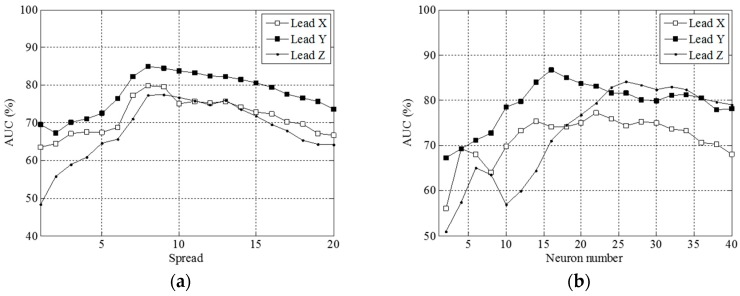
Graphs of (**a**) AUC vs. the spread parameters, keeping the neuron number fixed at 20; and (**b**) of AUC vs. the neuron number, keeping the spread parameter fixed at 10.

Exhibited in Figure 5d–f are the plots of the local performance indices against the number of neurons *M* in the X, Y and Z leads. Both TPA and sensitivity reached their maximum at *M* = 26, 32 and 34 in the X, Y and Z leads, respectively. We plotted a global performance index AUC against the spread parameter *σ* and the number of neurons *M*, in Figure 6a,b, respectively.

As indicated, Figure 6a,b give maximum values of 79.8%, 85.0% and 77.5% at *σ* = 8, 8 and 9 with *M* = 20 in the X, Y and Z leads, and of 77.3%, 86.7% and 84.1% at *M* = 22, 16 and 26 with *σ* = 10, respectively. We found AUC decreased with *σ* and *M* when it went beyond a certain threshold.

Table 1 shows the clinical performance of the AQR and time-domain VLP parameters. The performance of the VLP parameters was consistent with results of previous studies, reaching a maximum AUC of 87.5% (86.7%), a maximum TPA of 75% and a maximum sensitivity of 90.0%, but a specificity as low as 64.3%, in RMS40. By taking a linear combination of the VLP parameters, it is possible to attain consistency among local performance indices, that is, a TPA of 73.6%, sensitivity of 73.3% and specificity of 73.8%.

Table 1 also gives the respective AQR parameters along with the maximum TPA and sensitivity, keeping *M* constant at 20 (Figure 4) and *σ* constant at 10 (Figure 5). A maximum TPA of 83.0% and maximum sensitivity of 80.0% are visible in a Y lead case, AQR_Y(32, 10), reaching a specificity of up to 85.7% and an AUC of up to 81.1%, as well. In the AQR_Z(34, 10) case, we obtained a TPA of 81.9%, sensitivity of 76.6%, specificity of 85.7% and an AUC of 83.1%. In the AQR_X(26, 10) case, there was a TPA of 75.0%, sensitivity of 70.0%, specificity of 78.6% and an AUC of 74.4%.

Moreover, TPA, sensitivity and specificity can reach 80.0% or more with 20 neurons, taking a linear combination of 20 AQRs with *σ* ranging from 1 to 20, designated as AQR(20, 1:20), or with *σ* = 10 and taking a linear combination of 20 AQRs with the number of neurons *M* = 2, incremented by 2 each time, to 40, designated as AQR(2:40, 10).

Furthermore, a TPA of up to 93.1%, sensitivity of 86.7%, specificity of 97.6% and an AUC as high as 99.1% can be reached by a linear combination of 60 AQRs, i.e., AQR_X(20, 1:20), AQR_Y(20, 1:20) and AQR_Z(20, 1:20). Alternatively, 95.8% TPA, 90% sensitivity, 100% specificity and a 99.4% AUC can be attained by taking a linear combination of 60 AQRs, that is, AQR_X(2:40, 10), AQR_Y(2:40, 10) and AQR_Z(2:40, 10).

## 4. Discussion

### 4.1. The Proposed RBF Neural Network for Extracting Abnormal Intra-QRS Potentials

In this study, we synthesized a QRS complex using a non-linear RBF neural network, treating the so-called approximation error as a way to estimate the AIQP for patients at high risk of VT occurrence. We employed multiple Gaussian RBFs, each as a neuron, to approximate a QRS complex of interest. The spread parameter and the number of neurons are both key parameters in an RBF. All the RBFs had the same spread parameter and smoothness. AIQPs sharper than the RBF should be left out in the approximation error. Conceptually, our presented RBF neural network can be regarded as a special purpose filter for AIQPs with a passband determined by the spread parameter, once the number of neurons is specified. The center location of an RBF, i.e., the location where the maximum occurs, is found by means of an orthogonal least squares algorithm. Each time the orthogonal least squares algorithm is performed, the one with the maximum error reduction rate is selected from all the candidates as an added RBF center location, meaning that the greater the number of neurons, the smaller the approximation error will be. However, the use of an excessive number of neurons may overestimate the contribution from a normal QRS complex, but underestimate that from an AIQP. On the contrary, if the RBF neural network uses an inadequate number of neurons, it may overestimate the AIQP.

Because the optimum number of neurons is unknown and could vary among subjects, given a specified number of neurons and a spread parameter, the approximation error may contain the desired AIQP and the contribution of a QRS complex. A study [24] clearly indicated that the superposition of a normal QRS complex simulated by a low order ARMA(6, 6) parametric model, and an AIQP simulated as a white noise, in low frequency does reduce the accuracy and the reproducibility of AIQP estimates. Additionally, practical applications show reduced estimate accuracy owing to the high frequency components contained in a normal QRS complex [13,14]. In this study, with a simulated normal QRS complex and an AIQP simulated as 40–250 Hz colored noise, we found the approximation error included the wanted AIQP and the unwanted contribution from the normal QRS complex, both partially out of phase. In this circumstance, we saw a drop in the root-mean-square value of the approximation error in the presence of AIQP. With 50 simulated colored noise sources of the same bandwidth and the same root-mean-square value, we conducted up to 50 repetitions of AIQP estimates each for an RBFNN(20, 10) and an RBFNN(20, 12). Variations in the approximation error were seen across all repetitions, and, more importantly, in some cases a decrease in the root-mean-square value of the approximation error was actually observed in the presence of AIQP, resulting in a misjudgment. Hence, in clinical contexts one ought not to expect 100% accuracy from an AIQP. Nevertheless, comparison revealed a complementation between the existence and non-existence results of AIQP, determined by means of RBFNN(20, 10) and RBFNN(20, 12). We show that the misjudgment produced by RBFNN(20, 10) can be corrected by RBFNN(20, 12), and vice versa. In short, the use of either various numbers of neurons or various spread parameters is an effective means of improving the accuracy of the AIQP estimate.

Even though an RBF neural network is a nonlinear system, one finding of our research is that the approximation error varies directly in relation to the amplitude of a QRS complex. Hence, an increase in the approximation error may be ascribed to the presence of AIQP, the upscaling of a QRS complex, or even to both. In an attempt to rule out the unwanted error sources, we defined an AIQP-to-QRS ratio to normalize all the AIQP parameters by the root-mean-square value of a QRS complex, and as a way to remove the influence of the amplitude of the QRS complex on the AIQP estimate. Consistent with results of our previous studies, higher AQR parameters of signal-averaged electrocardiograms in the X, Y and Z leads are more clearly seen among VT patients than among normal subjects. With the same groups of subjects as in our previous studies, we have experimentally demonstrated that the normalization of UIQP by the root-mean-square value of a QRS complex leads to the higher UIQP ratios seen among VT patients compared to normal subjects [13,14].

### 4.2. Comparisons with Previous Studies

As in our previous studies [13,14], two linear prediction models, autoregressive moving average [13] and finite impulse response [14], have been employed to estimate the UIQP for diagnosing VT patients. UIQP refers to a collection of signals with abrupt slope changes distributed over a QRS complex, possibly including a sharp QRS waveform and a rapidly varying AIQP, and can be detected at locations where slope discontinuities occur by the prediction error of a linear prediction model. The main difference between the UIQP and AIQP analyses is that the UIQP analysis is to detect the slope changes induced by the AIQP [13,14], while the AIQP analysis is to extract the AIQP waveform [11,12,24]. As previously presented [11,12,24], a parametric modeling approach is based on the combination of multiple ARMA(2, 2) models, such that a QRS complex of interest can be analyzed in the DCT domain and AIQP can be estimated by means of modeling residuals. The output of an ARMA(2, 2) is represented as a time-domain biphasic signal. Essentially, the parametric modeling approach employs multiple biphasic signals in the time domain for the analysis of a QRS complex. The smoothness of the biphasic signal is described by its rising and falling slopes which are optimized in an ARMA(2, 2) model through a least squares approach. Because of the non-unified smoothness of biphasic signals, parametric modeling may have an output involving a mixture of a normal QRS complex and a rapidly varying AIQP. In contrast, all RBFs in the proposed RBF neural network had the same smoothness for synthesizing the QRS complex.

As in our previous studies [17], we used a maximum number of neurons, as many as the length of a QRS complex, to approximate a target QRS complex, in an attempt to minimize the approximation error, since each time instant in a QRS complex corresponds to an RBF center location. For this reason, an orthogonal least squares algorithm is not required to determine optimal RBF center locations. In contrast, the use of an excessive number of neurons in the RBF neural network presented here causes deteriorating clinical performance, as demonstrated in Figure 6. In other words, this maximum number strategy is not applicable to clinical diagnosis of patients with VT due to concerns about accuracy. In this study, we adopted various numbers of neurons and various spread parameters to test the diagnostic performance of an RBF neural network for VT patients. With an increase in the number of neurons or the spread parameter, the global diagnostic performances (i.e., AUC) of leads X, Y, and Z show a descending trend, as demonstrated in Figure 6. The spread parameter of around eight has the best global diagnostic performance, but the number of neurons for attaining the best performance was inconsistent for leads X, Y, and Z. In this study, the maximum TPA among all the individual AQR parameters was 83.3%, including the X, Y and Z cases. These results are superior to those achieved by a DCT-domain parametric modeling approach with 75.8%, 78.0% and 75% [11,12,24], as well as the 79.2% obtained using a finite-impulse-response linear prediction modeling approach [14]. Furthermore, looking at all the combinations of AQR parameters, we obtained a maximum TPA of 95.8% with 100% specificity and 90% sensitivity by taking a linear combination of 60 AQRs. This performance is higher than results of prior studies at 78.8%, 90.2% (but only 43.8% sensitivity) and 88.9% obtained using a DCT-domain parametric modeling approach [11,12,24], as well as the 83.3% achieved using a finite-impulse-response linear prediction model [14].

### 4.3. Limitations of the Study

The main limitation of the study is that the optimum number of neurons remains unknown and could vary among subjects. The AIQP may be overestimated or underestimated by the approximation error of the RBF neural network, and the effects of the AIQP estimation error may also vary among subjects. Hence the AIQP estimation error would reduce the diagnostic performance of AIQP. Although our results show that the linear combination of AIQP parameters using various numbers of neurons can improve diagnostic accuracy, further investigation with larger clinical populations is required to optimize the linear combination.

## 5. Conclusions

This study has successfully demonstrated the usefulness of a proposed RBF neural network for diagnosing VT patients at high risk of ventricular arrhythmias. Using RBFs with same smoothness it is possible to synthesize most of the QRS complex and extract the low-amplitude, high-frequency AIQPs using the approximation error. The estimated AIQPs are potentially sharper than RBFs, but may contain an estimation error arising from part of the normal QRS complex that cannot be synthesized accurately by the RBF neural network. The AIQP estimation error could cause clinicians to misjudge VT patients, and hence this limits the diagnostic accuracy of individual AIQP parameters of leads X, Y or Z. An important finding from our simulation study is that the misjudgment can be improved by combining multiple AIQP parameters estimated with various spread parameters and numbers of neurons. We defined an AIQP-to-QRS ratio that further corrected misjudgment arising from the effect of the inconsistent amplitudes of the QRS complex among subjects. Clinical results indicate that VT patients have higher AIQP-to-QRS ratios than normal subjects. The best total prediction accuracy of all individual AIQP parameters can reach 83.3%. A linear combination of 60 AIQP-to-QRS ratios greatly improves the total prediction accuracy, up to 95.8%. Future work will focus on reducing the estimation error of AIQP or on enhancing the accuracy of modeling the normal QRS complex to increase the clinical feasibility of using AIQP to diagnose VT patients.

## Figures and Tables

**Table 1:** Clinical performance of AQR and time-domain ventricular late potentials (VLP) parameters.

Parameters	Number of Parameters	SP (%)	SE (%)	TPA (%)	AUC (%)
Time-domain VLP parameters					
fQRSD	1	64.3	73.3	68.1	71.4
RMS40	1	64.3	90.0	75.0	86.7
LAS40	1	71.4	70.0	70.8	81.1
Linear combination of VLP parameters					
fQRSD + RMS40 + LAS40	3	73.8	73.3	73.6	83.8
Individual AQR parameters					
AQR_X(20, 12)	1	81.0	60.0	72.2	75.3
AQR_Y(20, 9)	1	90.5	73.3	83.3	84.6
AQR_Z(20, 8)	1	76.2	73.3	75.0	77.3
AQR_X(26, 10)	1	78.6	70.0	75.0	74.4
AQR_Y(32, 10)	1	85.7	80.0	83.3	81.1
AQR_Z(34, 10)	1	85.7	76.7	81.9	81.3
Linear combination of AQR parameters					
AQR_X(20, 1:20)	20	85.7	80.0	83.3	88.6
AQR_Y(20, 1:20)	20	92.9	80.0	87.5	93.6
AQR_Z(20, 1:20)	20	88.1	80.0	84.7	93.3
AQR_X(20, 1:20) + AQR_Y(20, 1:20) + AQR_Z(20, 1:20)	60	97.6	86.7	93.1	99.1
AQR_X(2:40, 10)	20	85.7	86.7	86.1	92.5
AQR_Y(2:40, 10)	20	83.3	80.0	81.9	89.4
AQR_Z(2:40, 10)	20	88.1	80.0	84.7	92.4
AQR_X(2:40, 10) + AQR_Y(2:40, 10) + AQR_X(2:40, 10)	60	100	90	95.8	99.4

AQR_*l* (*M*, *σ*) denotes the AIQP-to-QRS ratio estimated by an RBFNN with the neuron number *M* and the spread parameter *σ* in lead *l*, AQR_*l* (20, 1:20) denotes 20 AQR parameters estimated by an RBFNN with *σ* = 1, 2, …, 20, while keeping *M* constant at 20, and *l* represents the X, Y or Z lead, and AQR_*l* (2:40, 10) denotes another 20 AQR parameters estimated by an RBFNN with *M* = 2, 4, …, 40, while keeping *σ* constant at 10. SP, SE, TPA and AUC denote specificity, sensitivity, total prediction accuracy and the area under the receiver operating characteristic curve, respectively.
